# Supplementary material for: Systems analysis of intracellular pH vulnerabilities for cancer therapy
Source: Nat Commun. 2018 Jul 31;9:2997. doi: 10.1038/s41467-018-05261-x (PMC6068141; doi:10.1038/s41467-018-05261-x)
Supplement: Supplementary file 1 — Supplementary Information [file 41467_2018_5261_MOESM1_ESM.pdf]

Supplementary Information

Supplementary Figures

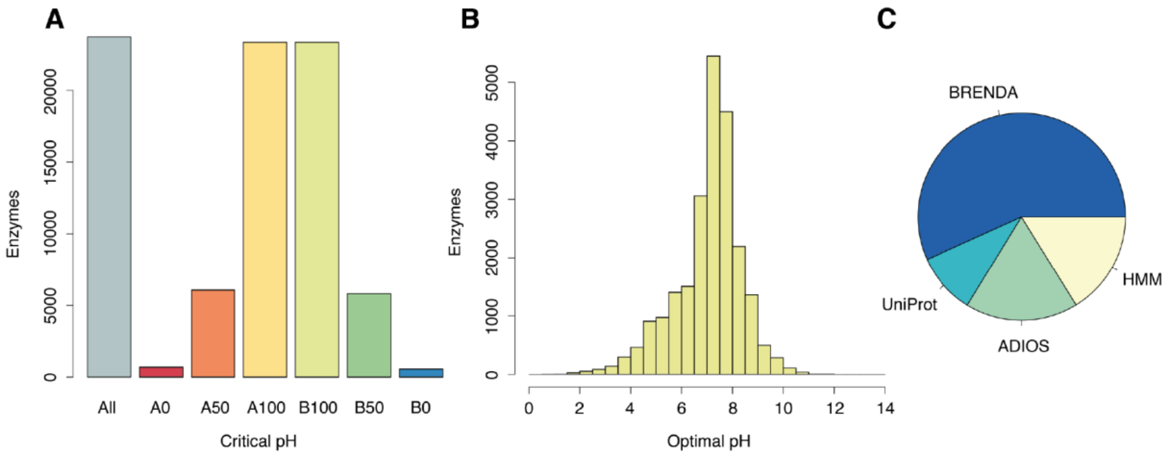

Supplementary Figure 1. Information extracted from BRENDA. (**A**) Number of enzymes for which experimental data were available from BRENDA, at each critical point (acidic and basic limits of 0%, 50% and 100% of activity). For most enzymes, the 100%-activity points were available. (**B**) Distribution of optimal pHs (average between 'A100' and 'B100') across all enzymes. (**C**) Sources of sequence annotation, being BRENDA annotation the priority, followed by UniProt, then ADIOS, and finally HMM search.

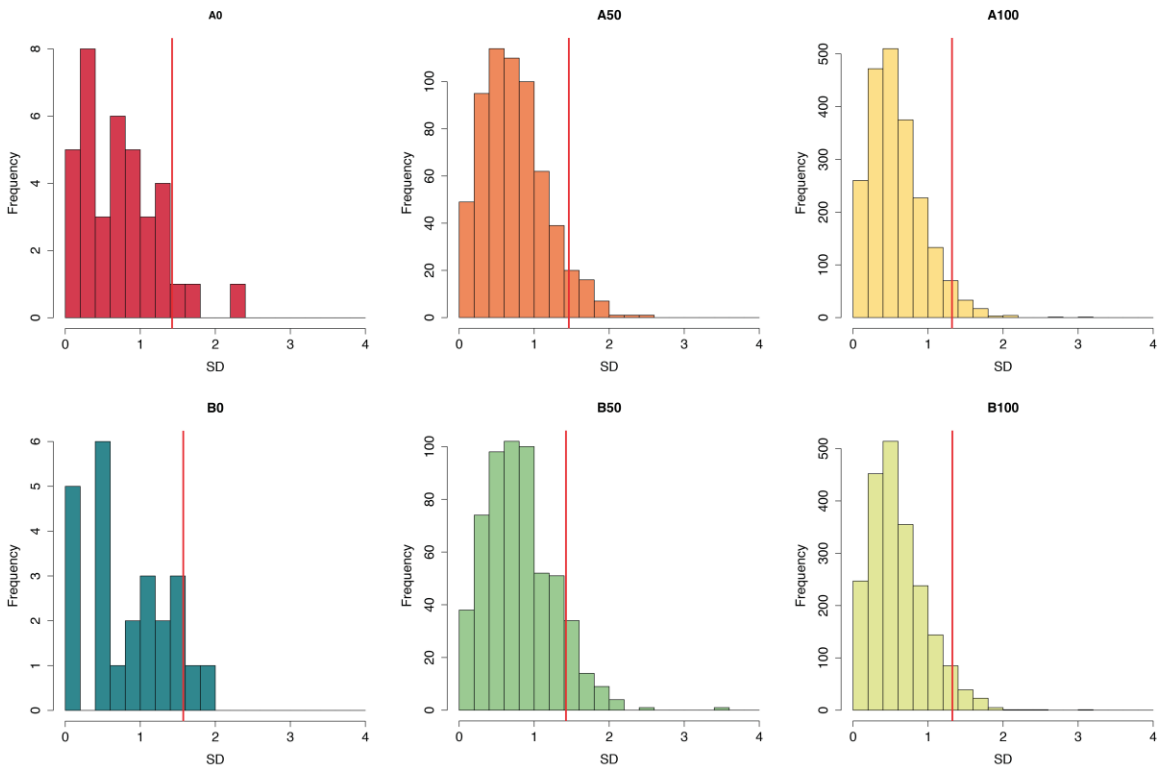

Supplementary Figure 2. Standard deviation (SD) of critical pH values within the same EC number (only ECs with at least three instances included). The red line denotes the background SD; *i.e.*, the global SD across *all* enzymes, without acknowledging EC information.

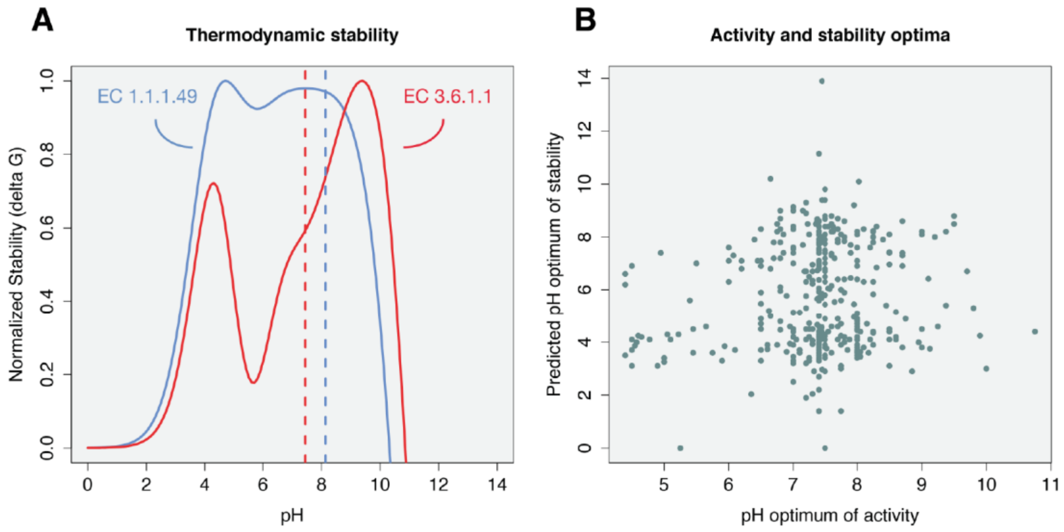

Supplementary Figure 3. Lack of correlation between predicted pH of optimal stability and activity. (**A**) Stability curves of two human enzymes for which 3D structures were available. Dotted lines indicate the experimental optima reported in BRENDA. (**B**) Calculated optima of stability *vs.* optima of activity.

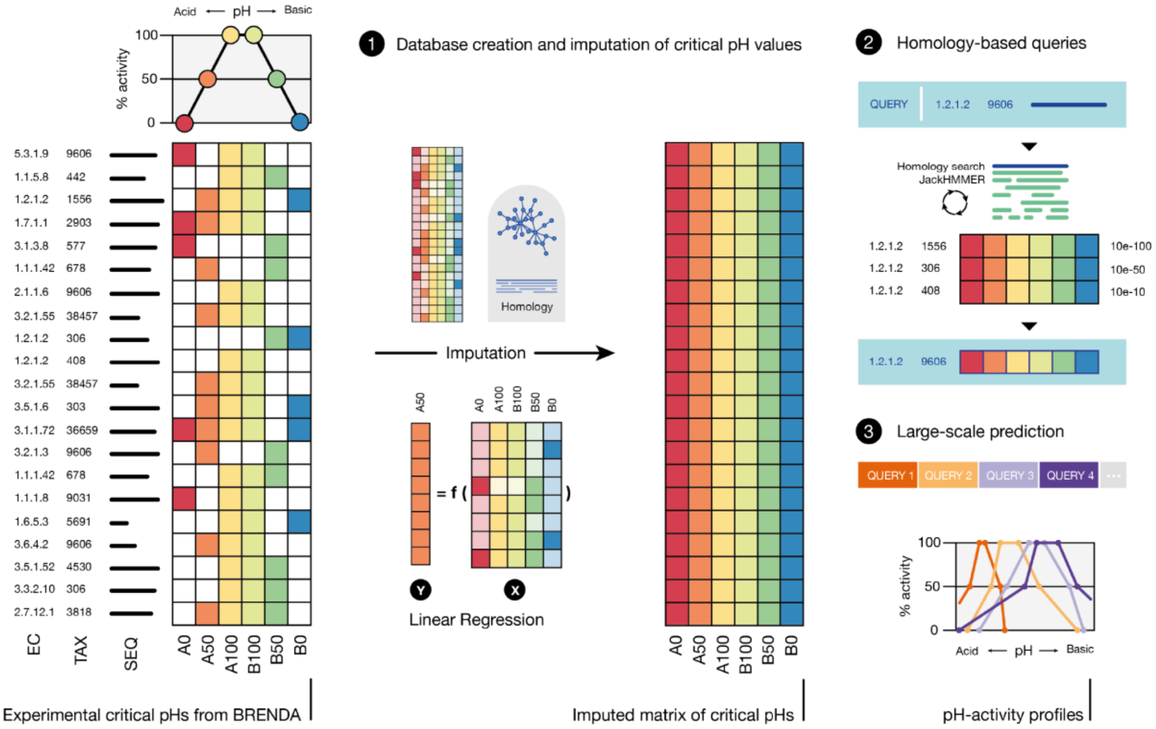

Supplementary Figure 4. Scheme of the pH-profiling method. (1) The missing data in the experimental database were imputed to obtain full activity profiles for each enzyme. For this, a preliminary matrix based sequence homology was first built. Then, this matrix was used to build linear regressors, whose predictions are used to impute the experimental matrix and yield a fully filled database of profiles. (2) To predict pH-activity profiles for a new enzyme, this database was searched using JackHMMER. (3) This querying procedure can be easily performed on a large scale.

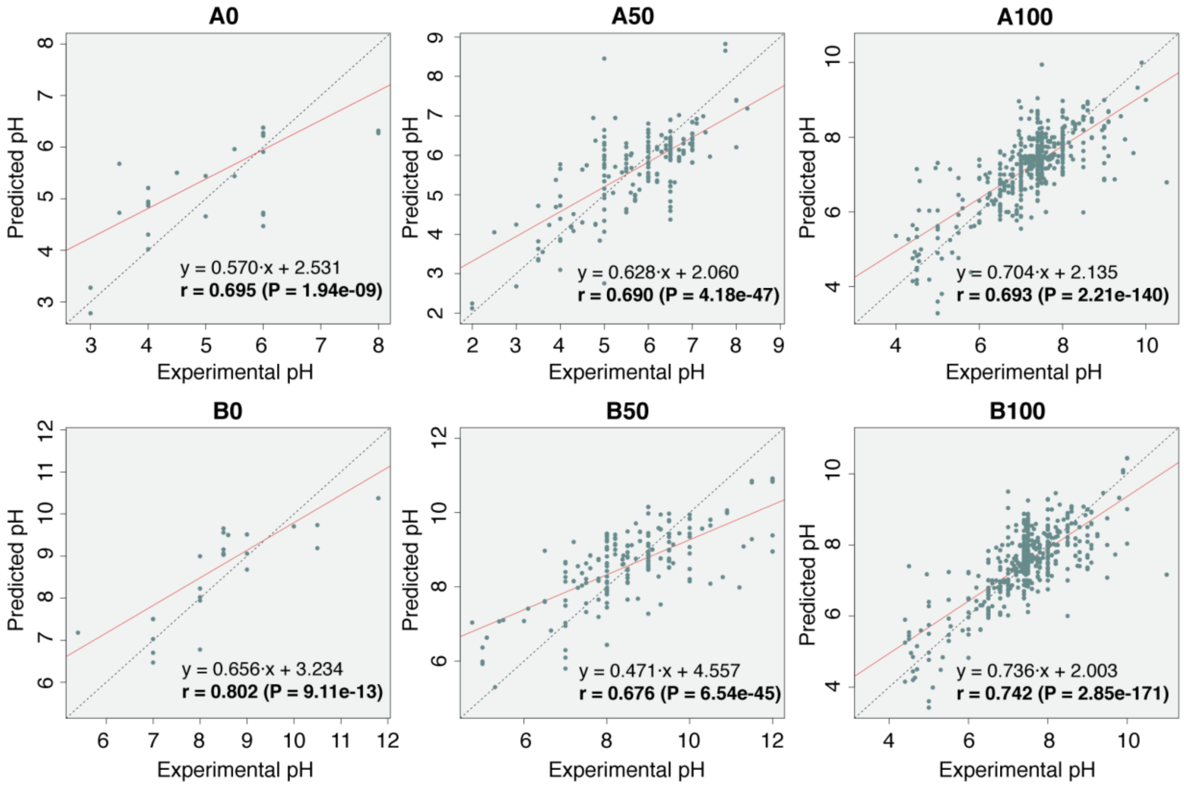

Supplementary Figure 5. Predicted *vs.* experimental critical pHs for the human dataset, after a 10-fold cross-validation. The points correspond to the predictions on the test sets. Pearson's correlation coefficients (r) are shown in the plots.

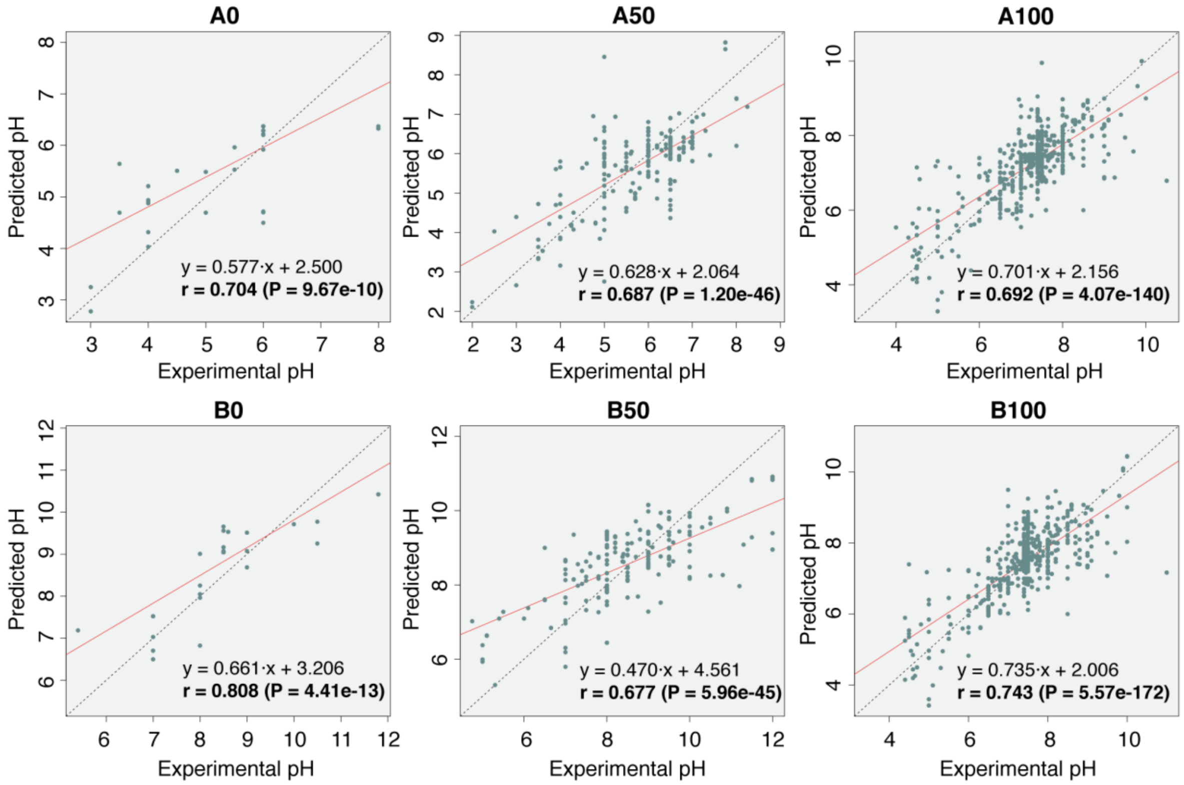

Supplementary Figure 6. Similar to Supplementary Figure 5, predicted *vs.* experimental critical pHs, when all human enzymes are removed from the training set.

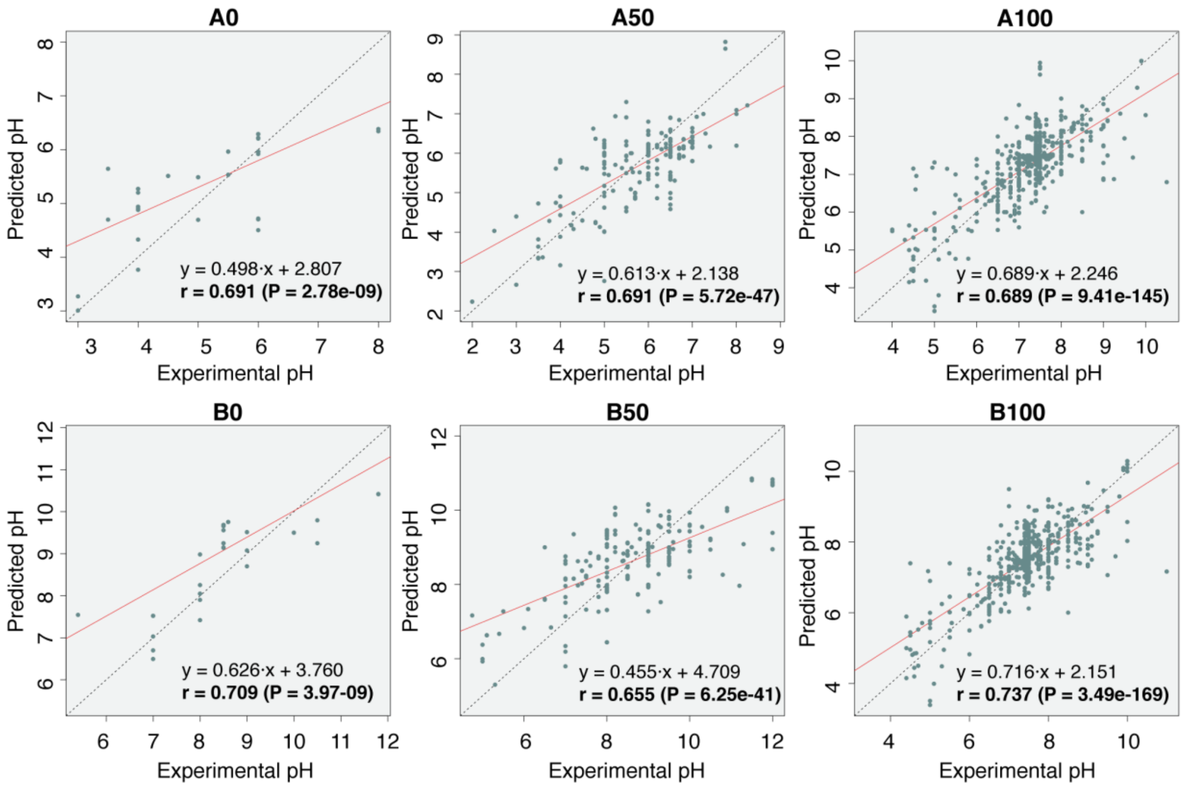

Supplementary Figure 7. Similar to Supplementary Figures 5-6, predicted *vs.* experimental critical pHs, when all human enzymes are removed from the training set, and EC number information is removed from the test set.

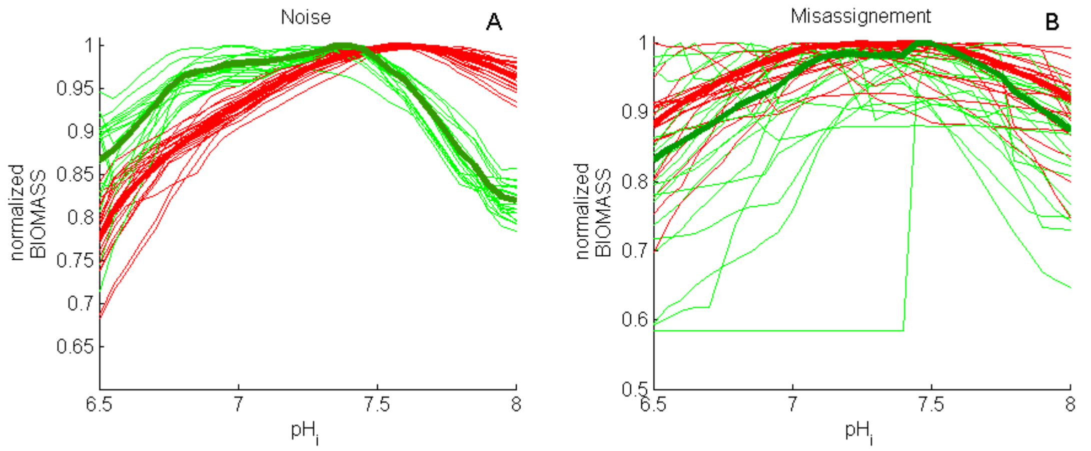

Supplementary Figure 8: Robustness analysis performed on a single cancer-normal cell-lines pair. **A)** the effect of perturbing the enzymes' pH-profiles by Gaussian noise (μ=0, σ=0.1) on the cellular proliferation of a cancer cell (red) and healthy cell (green) as function of pHi. Normalized biomass of 20 independent realizations of the Gaussian noise is shown in solid-thin curves. Solid-thick curves depict the average profiles. **B)** the effect of random (i.e., wrong) assignment of pH-profile to enzymes. Differences between cancer (red) and healthy (green) cells are lost, specifically at low-pHi. Solid-thin curves represent 20 independent realizations of random assignments. Solid-thick curves depict the average behavior.

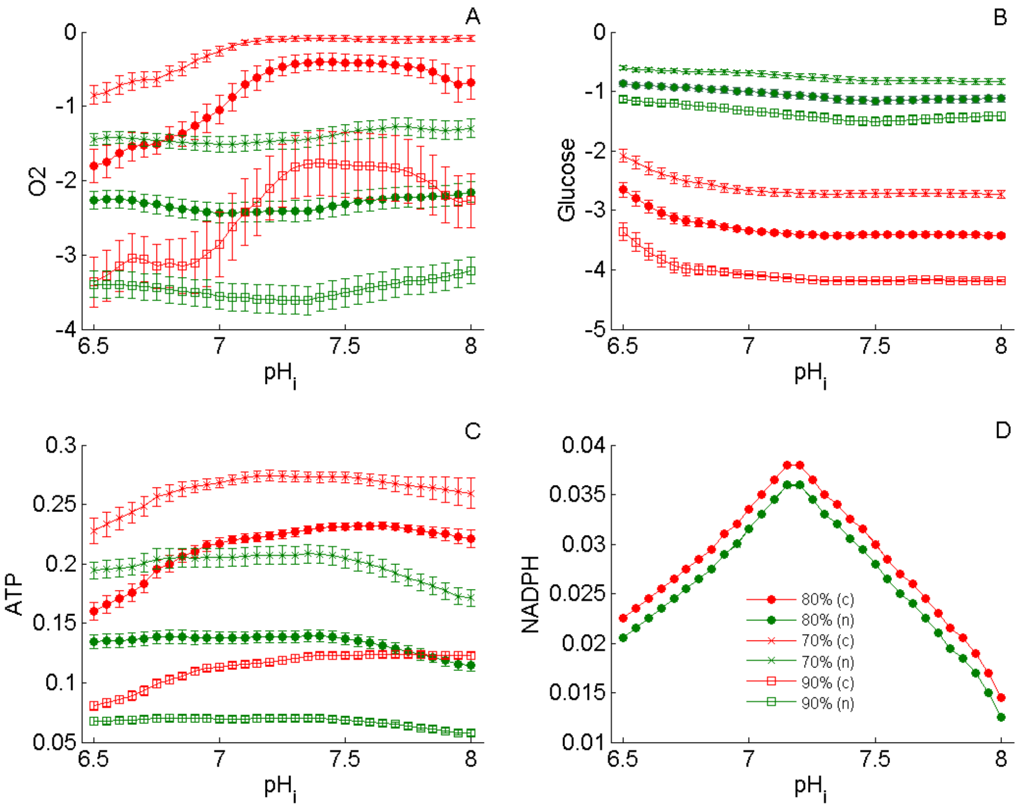

Supplementary Figure 9: Robustness analysis across the panel of cancer (red) and normal (green) cell-lines examined in figure 2 of main text, demonstrates that the behavior of key metabolites, *i.e.*, oxygen consumption rates **(A)**, glucose consumption rates **(B)**, ATP production rates **(C)** and NADPH production rates **(D)** hold for various choices of the constraint on the objection cellular function, *i.e.*, larger than X% of the FVA biomass maximum (see Methods). Shown are the behaviors in the range X = [70%-90%]. As depicted, this parameter only scales the absolute values of metabolite consumption/production but does not change the overall trends observed across the pHi and the evident differences between cancer and normal cells.

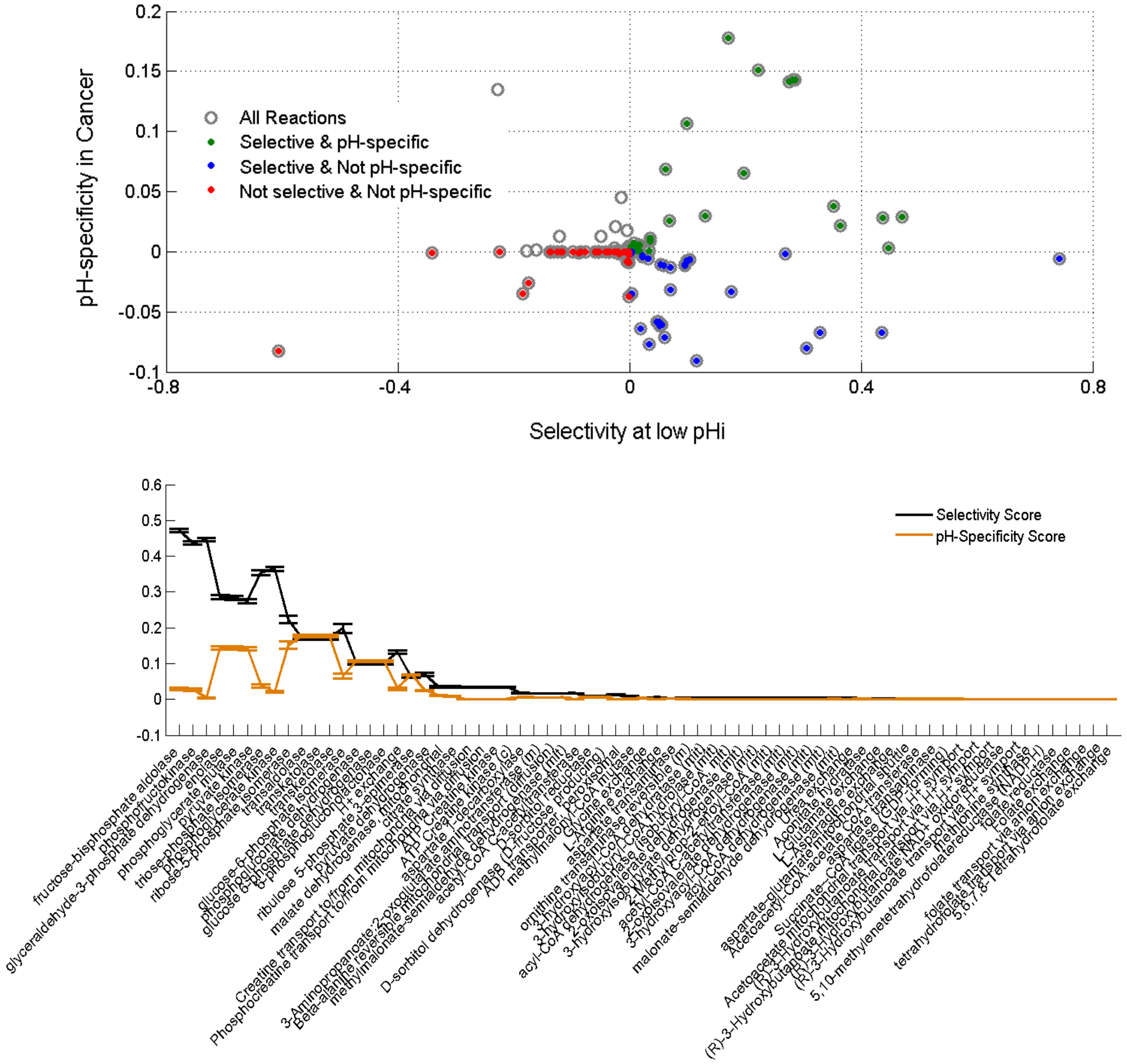

Supplementary Figure 10: Similar analysis to the one presented in Figure 2 of the main text (gene knockout), is shown here for each reaction knockout. Obviously genes which catalyze the same reaction, which are usually paralogs (such as *GAPDH* and *GAPDHS*) cannot be identified with in-silico single-gene knockout presented in Figure 2 of the main text. Nonetheless, the present figure demonstrates that they are identified by single-reaction knockout screening: glyceraldehyde 3-phosphate dehydrogenase (the reaction catalyzed by *GAPDH* and *GAPDHS*) is ranked third by its selectivity score and is also pH-specific.

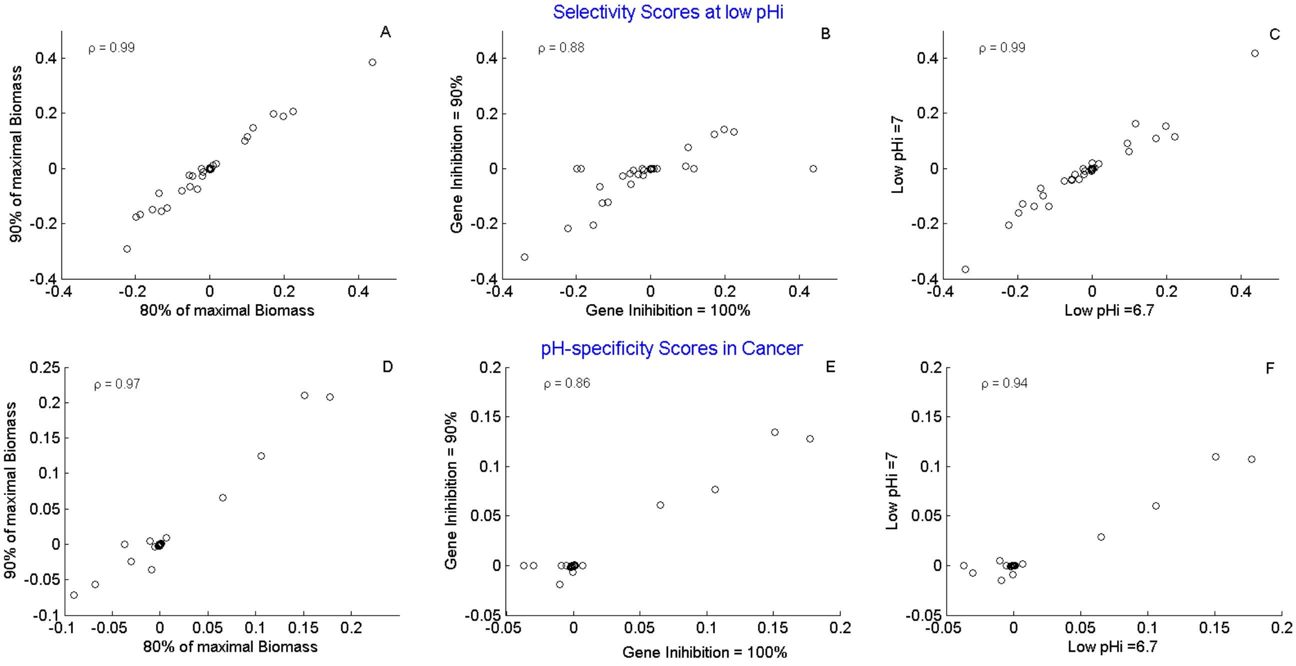

Supplementary Figure 11: Sensitivity of gene inhibition scores, selectivity (**A-C**) and pH-specificity (**D-F**) to the choice of parameters in the genome-scale metabolic models of cancer and normal cells. The correlation between the score is shown for: **A,D)** different choices of the constraint on the minimal percentage of the FVA-maximal biomass production [80%-90%]; **B, E)** different choices of the percentage of gene inhibition [90%-100%]; and **C, F)** the value of 'low' pHi chosen [6.7 -7]. ρ values are Pearson correlation coefficients.

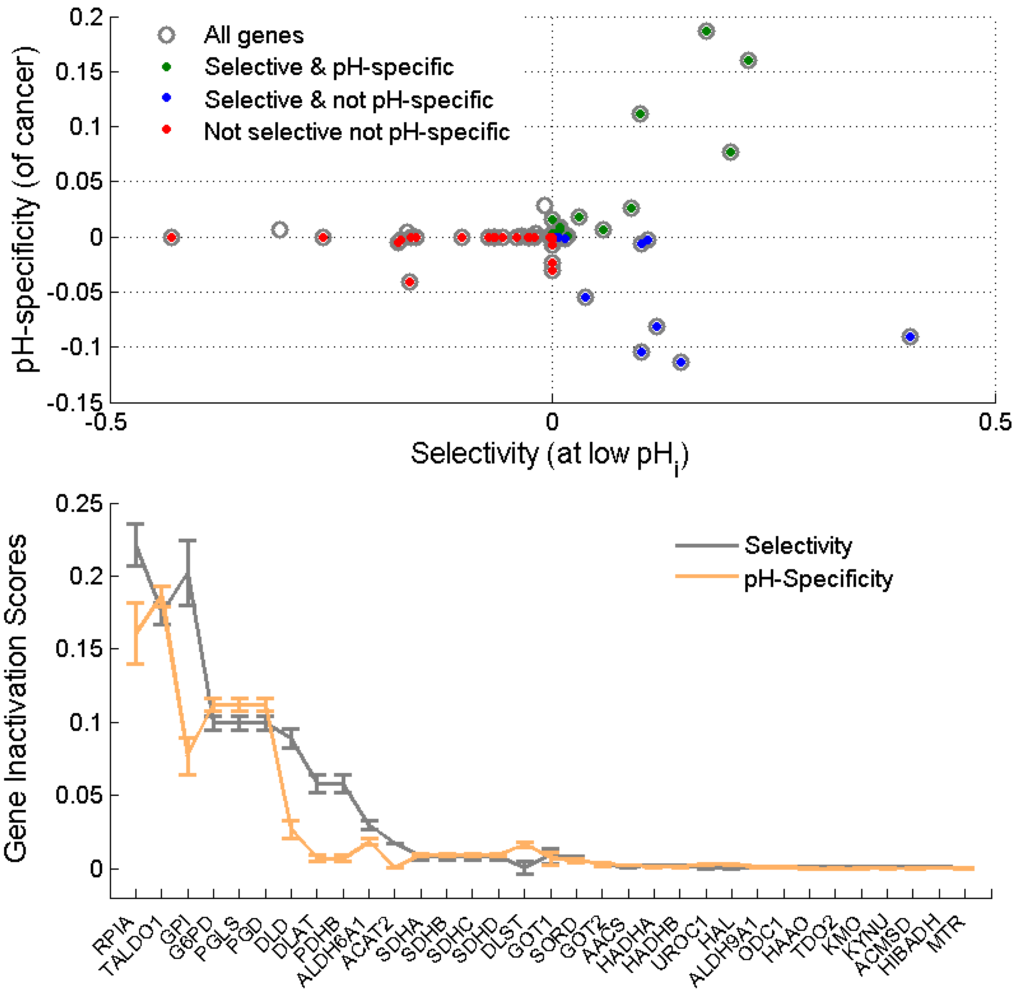

Supplementary Figure 12: Similar pH-specific and selective targets are identified when the flux bound correction (Methods) is applied to all enzymes in the model, as opposed to just the cytosolic enzymes (as in Figure 2 of the main text), demonstrating robustness of the identified targets. Note that when more constraints are applied (*i.e.*, restricting bound of all enzymes as in this figure) the metabolic networks are, as expected, more vulnerable and therefore more targets are identified. Hence, the results presented in the main text represent the least sensitive network to perturbation in pHi.

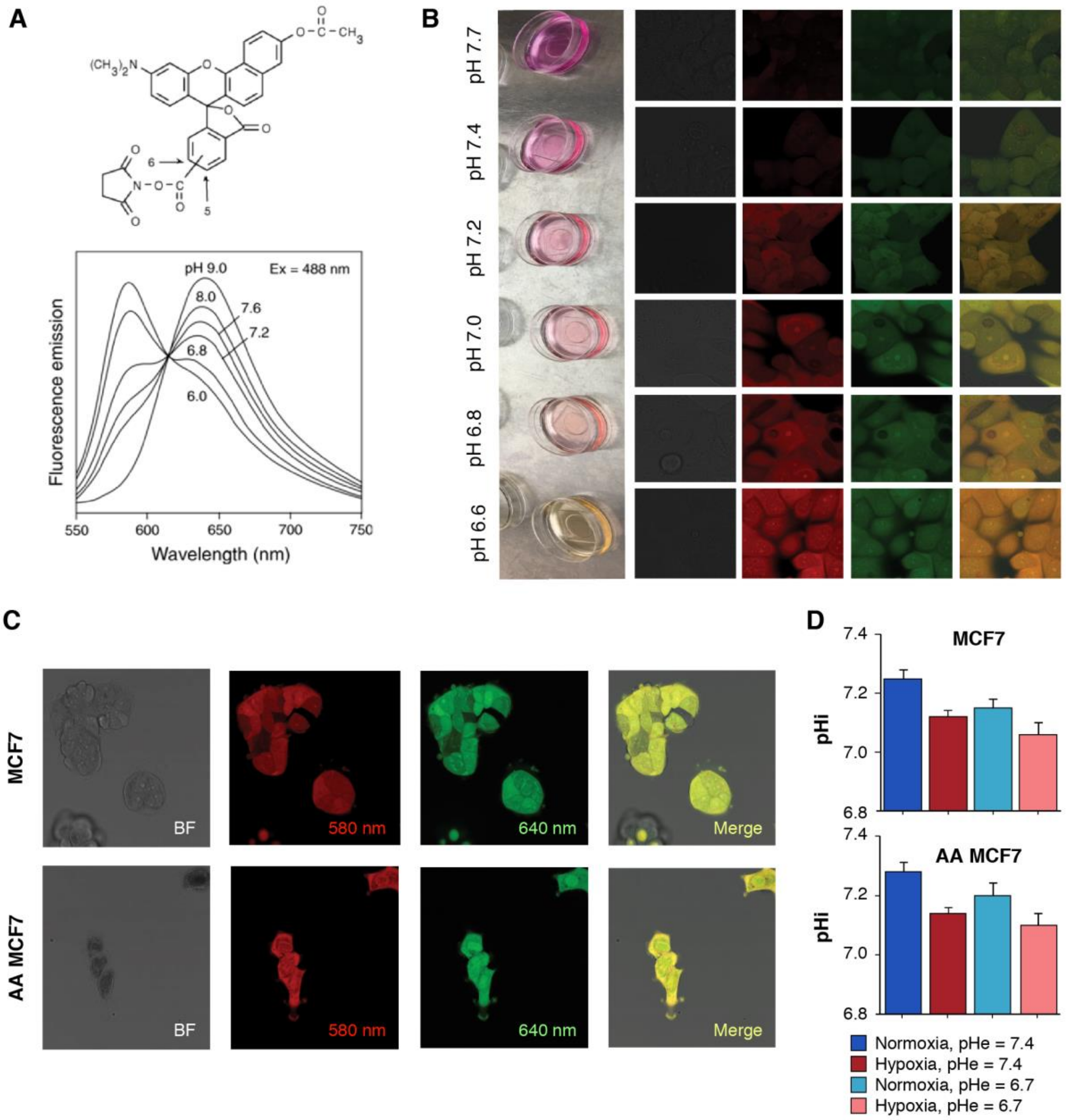

Supplementary Figure 13: Cancer cells intracellular pH measurement. **A)** the pH-sensitive probe molecule SNARF-1 and its pH-dependent emission spectra, taken from [https://www.thermofisher.com/order/catalog/product/S22801]. **B)** pH calibration of SNARF-1 in cells. Calibration of SNARF-1-loaded cells treated with 25μM Nigericin toxin at a target extracellular pH (pHe) removes light dispersion side effects from the analysis and allows accurate measurements of pKa. **C)** SNARF-1 pH fluorescent imaging of the two principal wavelengths in naïve and acid-adapted (AA) breast cancer MCF7 cells. **D)** pHi levels under different microenvironmental conditions (O_2 and pHe levels), estimated over at least thirty cells per condition, showing the week coupling between pHe and pHi. Acute hypoxia (0.1% O_2 for 15 min) reduces the pHi of cancer cells more dramatically. For these analyses, nucleus and lysosome are excluded and data are presented as mean and its error. The pHi analysis was done in at least 30 cells in each condition. Data are presented as the mean with the error of the mean as error bars.

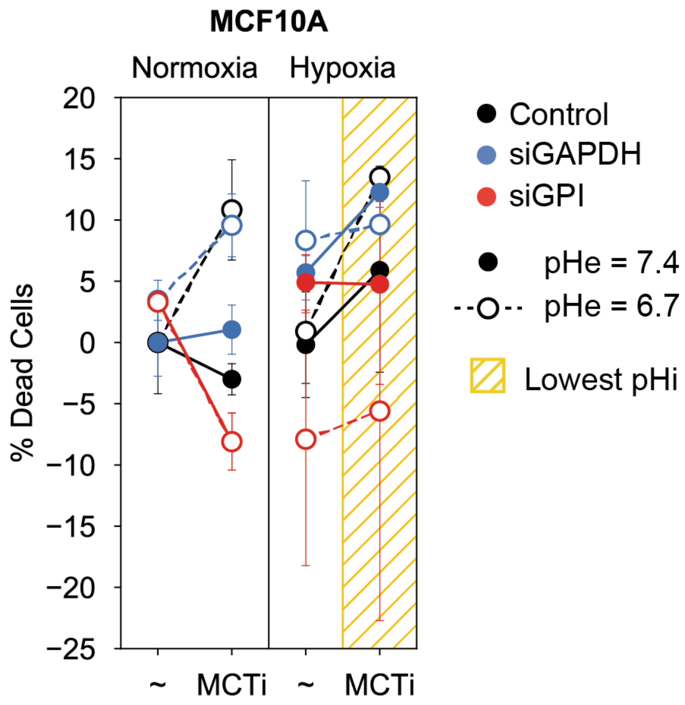

Supplementary Figure 14: Viability of normal epithelial MCF10 cells transfected with siRNAs targeting *GPI* and *GAPDH,* with or without (~) inhibition of *MCT1/2,* across all conditions examined. Here the strategy has only mild effect on cell survival; hence it is largely selective to cancer cells. See main text and discussion. Data is presented as mean and the error of the mean. The experiment was repeated three times with three replicates for each experiment. Data are presented as the mean with the error of the mean as error bars.

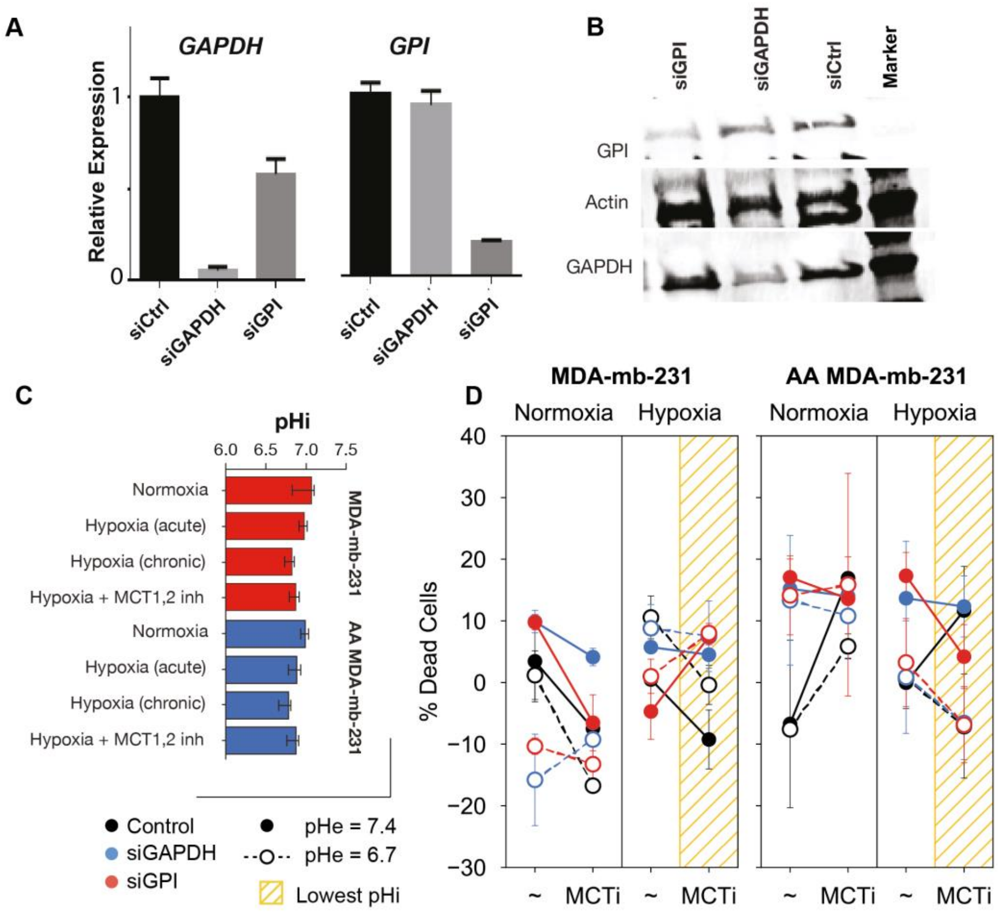

Supplementary Figure 15: Viability of MDA-MB-231 and AA MDA-mb-231 cells transfected with siRNA of *GPI* and *GAPDH*, with or without (~) inhibition of *MCT1/2*. **A-B)** Relative expression following targets inhibition (*left*) and corresponding western blots (*right*). qPCR was done in three replicates and the experiment was repeated three times. Data are presented as the mean with the error of mean as error bars. **C)** pHi was stable despite the applied perturbations, likely due to the expression of the MCT4 lactate transporter. For pHi analysis at least thirty cells was used. Data are presented as the mean with the error of mean as error bars. **D)** The strategy fails because low pHi is unattainable. See main text and discussion. Viability experiment is repeated three times with at least three replicates for each condition. Data are presented as the mean with the error of the mean as error bars.

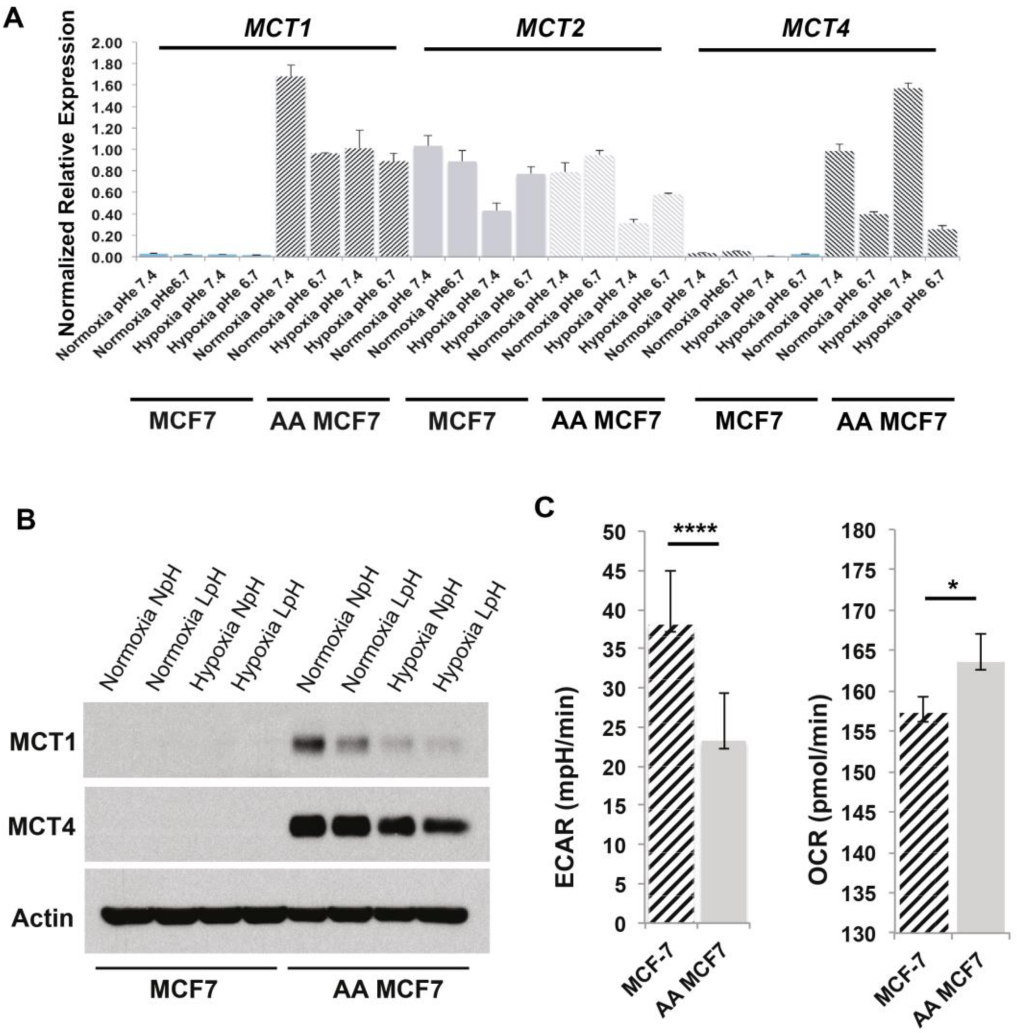

Supplementary Figure 16: **A)** Normalized relative expression of *MCT1*, *MCT2* and *MCT4*, under different microenvironmental conditions, in MCF7 naïve and acid-adapted (AA) breast cancer cells was determined by qRT-PCR. qRT-PCR experiment was done in three replicates and was repeated three times. Data are presented as the mean with the error of the mean as error bars. **B)** Western blots of *MCT1* and *MCT4* across the different conditions. **C)** Measurements of ECAR and OCR in naïve and acid-adapted MCF7 breast cancer cells. Seahorse experiment was done in six replicates and was repeated three times. Data were analyzed by unpaired t-test. *P*-value for ECAR is smaller than 0.0001 and the *P*-value for OCR is 0.0185. Data are presented as mean with SD as error bar.

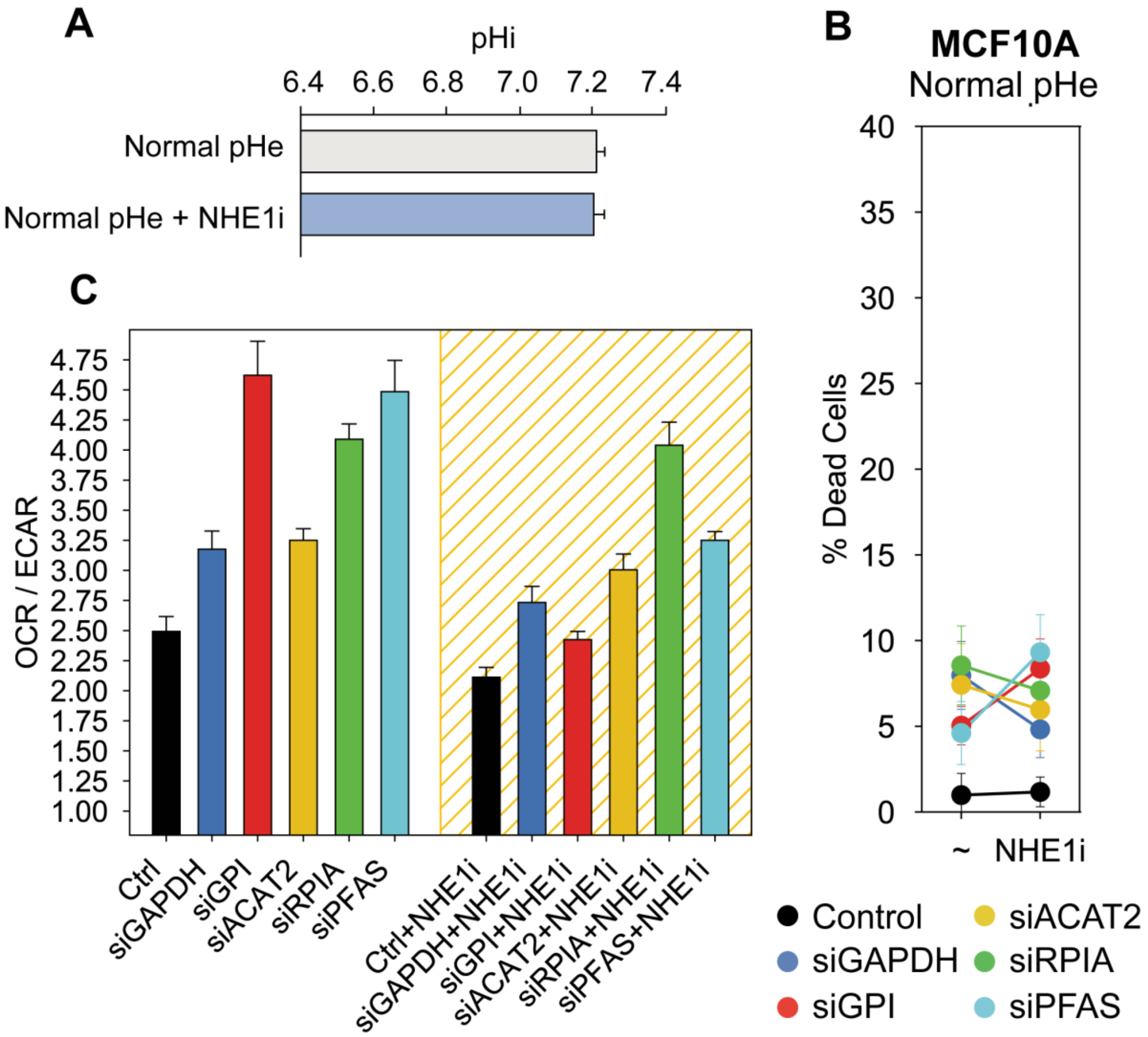

Supplementary Figure 17: A) The effect of extracellular pHe (Normal, 7.4 and low 6.7) and *NHE1* inhibition on the pHi of MCF10A cells. Data are presented as the mean with the error of the mean as error bars. **B)** Viability of normal MCF10A cells, transfected with siRNA inhibitors of the different metabolic targets, before and after the inhibition of *NHE1*. Only mild effects on viability are observed and cells are insensitive to *NHE1* inhibition, in their natural microenvironmental condition (i.e., normal pHe). Data are presented as the mean with the error of the mean as error bars. **C)** The ratios OCR/ECAR in MCF10A cells across conditions, as in Figure 5 of the main text. Lowering pHi did not lead to increased ratios as in MCF7 cancer cells (cf. Figure 5). The seahorse experiment was done in six replicates and the experiment was repeated twice. Data are presented as the mean with the error of the mean as error bars.

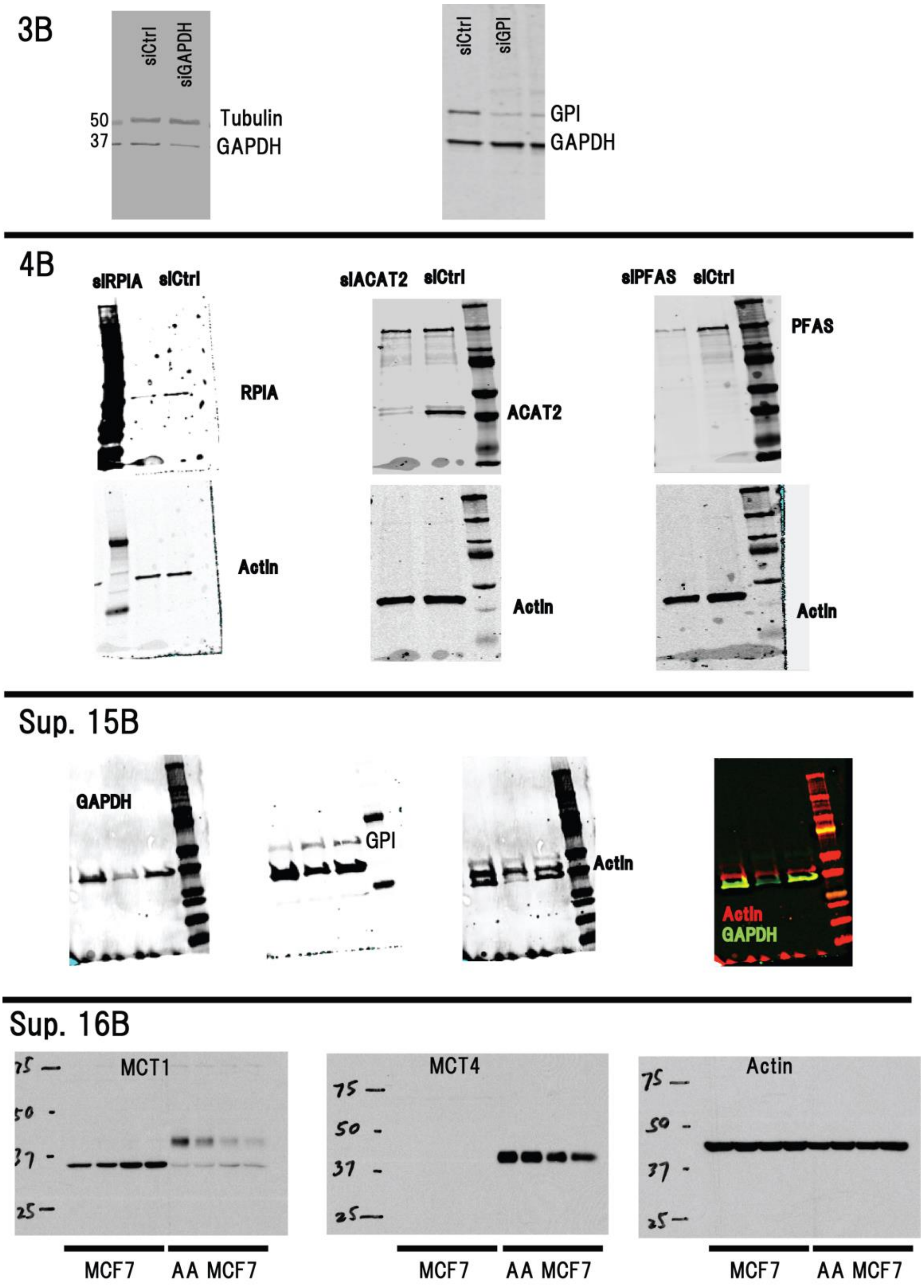

Supplementary Figure 18: All the uncropped western blots used to make the Figures in this manuscript, as indicated, for Figures 3B and 4B of the main text, and Supplementary Figures 15B and 16B, respectively.

Supplementary Methods

Background

Tight regulation of intracellular pH (pHi) is a fundamental property of living systems. Cells transduce energy through proton gradients and proton-coupled electron transfer reactions, and pH determines the charge state of weak acids and bases, affecting the physical and physiological properties of biomolecules such as proteins (*1*). For this reason, proteins need to be stable at the pH of the subcellular environment where they function. Protein pH stability depends on the amino acid composition and the 3D disposition of titratable groups. Recently, several computational methods have been developed to predict the pH of optimally stable proteins, requiring the calculation of the pKa of residues in folded and unfolded states, or the proportion of acid and basic residues in buried regions of the protein (*2*). However, pH fluctuations may affect more subtle phenomena, beyond stability, and directly affect protein function. For instance, enzymes have evolved to perform efficient chemical reactions involving electron transfers and proton translocations. Accordingly, there is a trade-off between reactivity and stability in the active site (*3*). Besides fold denaturation, loss of enzymatic activity at an aberrant pH can be thus ascribed to protonation states of catalytic residues, slight spatial modifications that hamper substrate or cofactor binding, or differential protonation of the substrate itself. Unlike pH stability calculations, physics-based methods are not yet able to integrate these factors and infer the one that limits activity, which many times depends on subtle phenomena, and predicting pH-activity profiles (*i.e.*, the full curve that relates enzymatic activity to pH) is a formidable task. These curves are usually bell-shaped around an optimal pH, although they can be also asymmetrical and, occasionally, multiple optima can be observed.

Fortunately, enzymes have been the matter of thorough biochemical study for several decades. A key parameter in enzymology is the pH of the buffer solution, which is optimized to identify the appropriate conditions for the assay. As a result of this process, pH activity curves, or at least some of their critical points, have been reported in numerous scientific publications. The BRENDA database [brenda-enzymes.org] is devoted to compiling these data and other information from the literature. Today, full or partial information on the pH sensitivity of enzymes is available for thousands of enzymatic functions in thousands of organisms.

The corpus of pH-activity information available in BRENDA allows us to test the hypothesis that homologous enzymes will respond similarly to changes in pH. Homologous proteins are similar in sequence and fold, and many times catalyze analogous reactions involving the same metabolites. Therefore, it is reasonable to postulate that pH fluctuations will affect homologs to a comparable extent, yielding similar pH activity curves. Here, based on the experimental data available in BRENDA, we embraced this hypothesis in the context of human metabolic enzymes. In particular, we

predicted the pH activity profiles of enzymes contained in the Recon1 reconstruction of human metabolism (Methods).

Experimental data

Raw pH-activity data: To compile an experimental dataset, we fetched all 'pH Optimum' and 'pH Range' records from BRENDA (July 2014). These included a total of 54,462 entries, of which 34,493 have comments edited by BRENDA curators.

Curation of pH records: In particular, 'pH Range' entries had comments on the extent of activity loss. These were the ones that required, most often, manual curation. Percentages of activity loss are not specified in BRENDA and were extracted from these comments. When no percentage value was provided, we assigned a 50% activity loss to descriptions referring to 'half activity', 'marked reduction' or similar, and 0% activity to cases like 'complete loss of activity' or 'no activity'. Curation of the optima was less laborious, although sometimes there were disagreements in the comments and the values presented by BRENDA. We disambiguated such cases by taking the value reported in the description.

Discretization of critical pHs: To delineate activity profiles that were suitable for machine learning, we defined six critical pH points, namely 'A0', 'A50', 'A100', 'B100', 'B50', and 'B0'. These correspond to the acidic (A) and basic (B) limits of 0%, 50%, and 100% of activity. Accordingly, activity values below 25% were rounded to 0%, and values between 25% and 75% were approximated to 50%. Activities above the 75% were set to 100% if no optima values were available. Regarding the 100%-activity points, when a single optimum was reported (as was most commonly the case) we assigned the same value to 'A100' and 'B100'. Often, BRENDA contained several records per enzyme. To minimize the impact of outliers, the median was calculated in these cases. If more than one optimum were reported, we defined a broad peak limited by the interquartile range.

In Supplementary Figure 1 we present global statistics of the database that we compiled. In total, we collected 23,658 enzyme entries, corresponding to 4,766 functions (4th EC level) in 4,521 organisms. Notably, 926 records corresponded to human enzymes, and 3,902 to mammals. As shown in Supplementary Figure 1A, for the vast majority of these cases we had data on the optimal pH, where half-activity points were less frequent, and complete loss of activity points was rare. Supplementary Figure 1B shows that these experimental values spanned a wide range of pH values.

Sequence annotation: Additionally, we assigned sequences to as many BRENDA enzymes as possible. Often, BRENDA itself provided sequence annotations and, in other cases, this information was extracted from UniProt if the BRENDA organism could be mapped to the NCBI taxonomy. Most

of the remaining entries were obtained by collecting species proteomes from UniProt, and then running ADIOS on these proteomes to assign EC numbers (*4*). Similarly, the remaining cases were completed by building HMM profiles across the full EC annotation in UniProt (*5*), and then running HMMSearch with default parameters against the proteomes (this annotation procedure is analogous to that used by BRENDA annotators, where sequences are often assigned based on BLAST results). As shown in Supplementary Figure 1C, we could assign sequences to most of the enzymes, two-thirds of them coming directly from BRENDA or from UniProt.

Proof of principle

Homologous enzymes have similar critical pH values: As noted above, homologous proteins were hypothesized to have similar pH activity curves. Supplementary Figure 2 validates this hypothesis, where enzymes that perform the same function (same EC number) have similar pH optima ('A100', 'B100') and their activities decay equally as one deviates from these optima ('A50', 'A0', 'B50', 'B0'). Note, also, that best precision was achieved for 100%-activity points.

As expected, in the database we observed a very strong correlation between function and sequence homology. Setting a homology threshold of E-value 1e-4, there was a strong enrichment of homologous sequences among enzymes with the same EC number (odds ratio of a right-tailed Fisher's exact test of 995.3, P-value < 10^{-323}). Overall, this confirms that sequence, function and pH profiles are tightly related.

Predicted pHs of optimal stability do not correlate with experimental activity optima: An alternative approach is to use pH-stability curves to estimate pH activity profiles. Others have suggested that a certain degree of correlation exists between the optima of stability and activity (*3*). To test this notion, we used the well-established PROPKA tool (v3.1) to calculate the pH-stability curves based on 3D structures. Supplementary Figure 3A shows that these stability curves, based on pKa titration of ionizable residues, are difficult to match with experimental optima of activity. In general, there was no clear correlation between calculated optimal stabilities and pHs of highest activity (Supplementary Figure 3B). Moreover, stability curves were not useful to predict the 50% and 0% critical points.

Database of pH-profiles

Given that, to date, no physics-based method can systematically predict pH-activity curves, we capitalized on the observation that homologous sequences have, in general, similar pH-profiles (Supplementary Figure 2). A schema of the method that we developed is provided in Supplementary Figure 4. In brief, gaps in the experimental database were filled to have a more complete database of profiles ('A0', 'A50', 'A100', 'B100', 'B50', 'B0') that could be later queried. To this end, missing

critical pH values were predicted largely based on other critical points, and knowledge on homologous enzymes. The net result was a database containing full pH-activity profiles that were a mixture of experimental and predicted values. Details of the method are explained below:

Imputation of critical pH values: As is evident in Supplementary Figure 1, most often all six critical pH values were not experimentally available for a given enzyme. In BRENDA, optimal (100%-activity) pHs are usually reported but reports of 50% and 0% activities are much rarer. To predict these missing points we built predictive models as follows:

I. A preliminary matrix containing values based on close homologs was filled (rows: enzymes; columns: critical pHs). For this, we previously constructed a network of enzymes in BRENDA where two enzymes were connected if they had sequence relatedness (computed with JackHMMER, with default parameters, E-value cutoff of 10^{-4}). Then, to complete each row (*i.e.*, each enzyme) we identified the closest homologs in the network with available experimental data, and calculated the weighted median; weights corresponded to the –log E-value.
II. After this homology-based imputation step, we performed a simple linear regression for each of the six critical pH points. To predict, 'A50', for instance, we used 'A0', 'A100', 'B100', 'B50', and 'B0' as variables from the previous matrix (I).
III. Finally, further fine tuning of the pH values (first or second decimals) could be obtained by up-weighting columns such as 'A100' and 'B100', where experimental data were more abundant and therefore homology-based values were more reliable. In particular, we readjusted those predictions that had a homology-based value within +/- 1 pH units of the predicted one. In this readjustment, we simply weighted by the proportion of experimental data in the column, from no influence (no weight) of the homology-based value in absence of experimental data, to the average with the predicted one in the hypothetical case of a fully experimental column.

Internal validation of the regressions: We performed a 10-fold cross-validation of each of the regressors, obtaining R-squared values of ~0.6-0.8, which were slightly improved after the homology-based readjustment (III).

Querying the pH-profile database

Once the pH-profile database was completed, human enzymes of interest were screened against this database (Supplementary Figure 4). For this, genes in Recon1 were mapped to UniProt identifiers using UniProt's IdMapping tool. EC numbers were also extracted from UniProt. In total, EC numbers

were assigned to 1,444 of the 1,905 genes in Recon1. Using the pH-profile database, we tried to infer the pH-profile of these enzymes.

Functional matching: The first step in the search was to find records having the same EC number as the query enzymes. Please note that, even when we found the exact human record (which was the case for ~500 enzymes), this doesn't mean that the full experimental profile was available. Rather, most of the time it meant that the experimental optimum was available, but not the rest of critical points, which were predicted in the imputation step. Overall, 80.5% of the human enzymes in Recon1 were found in the database, and for another 19% we found records with the same EC number in other species. Only for 0.5% of the human enzymes lacked sequence data, and in these cases it was necessary to find homologs in the database.

Homology-based weighting: When several records from different species were matched, and if sequences were available, a JackHMMER search was performed to assign E-values of homology. Then, each critical point was calculated as a weighted median; weights corresponding to the –log E-value (upper limit of 200). Preference was given to enzymes with more abundant experimental data by exclusively selecting them if they had a –log E value above 100.

Using this procedure we derived, for almost 1,500 enzymes in Recon1, the acidic and basic limits of none, half, and full activity; i.e., their 'A0', 'A50', 'A100', 'B100', 'B50', and 'B0' critical pHs.

Validation

We extensively validated the pH-activity profiles of the Recon1 enzymes by submitting our method to three cross-validation protocols of increasing merit:

Standard 10-fold cross-validation: To ensure complete independence between training and test sets in the cross-validation, and to avoid over-fitting the data, training and test sets were split from the raw experimental data that we compiled from BRENDA; *i.e.*, before the homology-based imputation step. In addition, since more than one gene in Recon1 could have the same EC number, all human records in the training set were removed that had EC codes represented in the test set. Results of the 10-fold cross-validation are shown in Supplementary Figure 5.

Removal of human enzymes: In addition, a stringent validation was performed by removing *all* human enzymes from the initial dataset. Results are shown in Supplementary Figure 6.

Removal of human enzymes and EC number information: Finally, an even more stringent validation was performed, where in addition to removing all human enzymes, EC information was excluded

from the search. Here, in practice, all predictions were based on the JackHMMER search, without functional (EC) supervision. Supplementary Figure 7 shows that the method continues to perform well in this case.

Supplementary References

1. Garcia-Moreno B (2009). **Adaptations to proteins to cellular and subcellular pH**. Journal of Biology, 8:98.
2. Alexov E (2004). **Numerical calculations of the pH of maximal stability**. *Eur J Biochem*, 271, 173-185.
3. Talley K & Alexov E (2010). **On the pH-optimum of activity and stability of proteins**. *Proteins*, 73:2699-2706.
4. Kunik V, Meroz Y, Solan Z, Sandbank B, Weingart W, Ruppin E, Horn D (2007). **Functional Representation of Enzymes by Specific Peptides**. *PloS Comp. Biol.*, 3(8):e167.
5. Tian W, Arakaki AK, Skolnick J (2004). **EFICAz: a comprehensive approach for accurate genome-scale enzyme function inference**. *Nucleic Acids Res*, 32(21):6226-39.
