# Supplementary material for: Systems analysis of intracellular pH vulnerabilities for cancer therapy
Source: Nat Commun. 2018 Jul 31;9:2997. doi: 10.1038/s41467-018-05261-x (PMC6068141; doi:10.1038/s41467-018-05261-x)
Supplement: Supplementary file 3 — Description of Additional Supplementary Files [file 41467_2018_5261_MOESM3_ESM.pdf]

Description of Additional Supplementary Files

File Name: Supplementary Data 1

Description: This XLSX file contains the list of human metabolic enzymes in RECON-1 (n=1905) and their respective inferred pHi profiles, which were used for the analyses described in our study.

File Name: Supplementary Software

Description: Contains: (i) the Matlab files and the algorithm that integrates pH-profiles into genome-scale metabolic models; (ii) the models used in our study; and (iii) analysis scripts that fully reproduce the in silico results. Supplementary Software is provided as a ZIP file (CODE.zip).
