# Supplementary material for: Systems analysis of intracellular pH vulnerabilities for cancer therapy
Source: Nat Commun. 2018 Jul 31;9:2997. doi: 10.1038/s41467-018-05261-x (PMC6068141; doi:10.1038/s41467-018-05261-x)
Supplement: Supplementary file 5 — Supplementary Software [file 41467_2018_5261_MOESM5_ESM.zip › CODE/Dataset S2_READ_ME.docx]

**Dataset S2**

The data (in CODE.zip) contains Matlab files of the models used in this study, the algorithm to integrate pH profiles into genome-scale metabolic models and perform an *in-silico* analysis, as well as (pre-processed) output results of the algorithm, including analysis scripts that reproduce the main results (**Figure 2**).

**Extract the folder in your computer. It contains two data files:**

**1) INPUT.mat**

Contains the PRIME models of 8 NCI-60 cancer cell lines (nci_CL_NAME) and the 12 HapMap normal cell lines (hm_CL_NAME), inferred pH-profiles of the metabolic enzymes in RECON-1 (ph_weights) across the range of pH tested (ph_vals). The model structures contain information of the gene, reaction and the gene-reaction mapping as well as other information. This information is identical for all models. They differ only in their lower and upper bounds of the reactions (model.lb, model.ub).

**2) OUTPUT.mat**

These are the results of the algorithm described below in two structures: res_nci_ko and res_hm_ko.

**To follow the algorithm, you need Matlab and tomlab toolbox. Do the following**

1. Open Matlab, go to the CODE directory that you extracted and make sure tomlab is running (run startup.m, if needed).

2. Load INPUT.mat

2. Open the file MAIN_ALGO.m and follow the instructions and steps. There is “USE” commands, copy-paste them into the command line and run. Simply follow the steps. There are comments and explanations for each step.

Once applied to all models, it will reproduce **Output.mat**

**To follow the analysis and reproduce the results you need only Matlab. Do the following:**

1. Open Matlab, go to the CODE directory that you extracted.

2. Load INPUT.mat

3. Load OUTPUT.mat

4. Open the file MAIN_ANALYSIS.m and follow the instructions and steps. There is “USE” commands, copy-paste them into the command line and run. This script will generate the main results in Figure 2 (and more).
